# A novel consortium of *Lactobacillus rhamnosus* and *Streptococcus thermophilus* for increased access to functional fermented foods

**DOI:** 10.1186/s12934-015-0370-x

**Published:** 2015-12-08

**Authors:** Remco Kort, Nieke Westerik, L. Mariela Serrano, François P. Douillard, Willi Gottstein, Ivan M. Mukisa, Coosje J. Tuijn, Lisa Basten, Bert Hafkamp, Wilco C. Meijer, Bas Teusink, Willem M. de Vos, Gregor Reid, Wilbert Sybesma

**Affiliations:** Yoba for Life Foundation, Hunzestraat 133-A, 1079 WB Amsterdam, The Netherlands; Micropia, Natura Artis Magistra, Plantage Kerklaan 38-40, 1018 CZ Amsterdam, The Netherlands; Department of Molecular Cell Biology, VU University Amsterdam, De Boelelaan 1085, 1081 HV Amsterdam, The Netherlands; TNO Microbiology and Systems Biology, Zeist, The Netherlands; CSK Food Enrichment, Ede, The Netherlands; Department of Veterinary Biosciences, University of Helsinki, Agnes Sjöberginkatu 2, 00790 Helsinki, Finland; Department of Food Technology and Human Nutrition, Makerere University, Kampala, Uganda; Department of Bacteriology and Immunology, RPU Immunobiology, University of Helsinki, Helsinki, Finland; Laboratory of Microbiology, Wageningen University, Wageningen, The Netherlands; Canadian Centre for Human Microbiome and Probiotic Research, Lawson Health Research Institute, London, ON Canada; Division of Urology, Department of Microbiology and Immunology, Department of Surgery, Western University, London, ON Canada

**Keywords:** Enrichment, *Lactobacillus rhamnosus* GG, *Lactobacillus rhamnosus* yoba 2012, *Streptococcus thermophilus* C106, Bacterial fermentation, Consortium, Yoghurt, Functional foods, Fermented foods

## Abstract

**Background:**

The lactic acid bacterium *Lactobacillus rhamnosus* GG is the most studied probiotic bacterium with proven health benefits upon oral intake, including the alleviation of diarrhea. The mission of the Yoba for Life foundation is to provide impoverished communities in Africa increased access to *Lactobacillus rhamnosus* GG under the name *Lactobacillus rhamnosus* yoba 2012, world’s first generic probiotic strain. We have been able to overcome the strain’s limitations to grow in food matrices like milk, by formulating a dried starter consortium with *Streptococcus thermophilus* that enables the propagation of both strains in milk and other food matrices. The affordable seed culture is used by people in resource-poor communities.

**Results:**

We used *S. thermophilus* C106 as an adjuvant culture for the propagation of *L. rhamnosus* yoba 2012 in a variety of fermented foods up to concentrations, because of its endogenous proteolytic activity, ability to degrade lactose and other synergistic effects. Subsequently, *L. rhamnosus* could reach final titers of 1E+09 CFU ml^−1^, which is sufficient to comply with the recommended daily dose for probiotics. The specific metabolic interactions between the two strains were derived from the full genome sequences of *L. rhamnosus* GG and *S. thermophilus* C106. The piliation of the *L. rhamnosus* yoba 2012, required for epithelial adhesion and inflammatory signaling in the human host, was stable during growth in milk for two rounds of fermentation. Sachets prepared with the two strains, yoba 2012 and C106, retained viability for at least 2 years.

**Conclusions:**

A stable dried seed culture has been developed which facilitates local and low-cost production of a wide range of fermented foods that subsequently act as delivery vehicles for beneficial bacteria to communities in east Africa.

**Electronic supplementary material:**

The online version of this article (doi:10.1186/s12934-015-0370-x) contains supplementary material, which is available to authorized users.

## Background

Diarrheal diseases and associated malnutrition remain a leading cause of mortality and morbidity of children in low-income countries in sub-Saharan Africa [1]. Beneficial bacteria, such as *Lactobacillus rhamnosus* GG are known to contribute to the reduction of the overall incidence of symptomatic rotavirus associated diarrhea, as reported in a recent meta-analysis [2]. At present, such effective probiotics are not available in sub-Saharan Africa or not affordable to the poor. Other means of ingesting beneficial microbes include fermented foods, of which various forms have a long history in Africa and are broadly acknowledged to contribute to a healthy life style and gastrointestinal wellbeing [3, 4]. Unfortunately, traditional food processing is often hampered by spoilage; in Uganda alone, 27 % of the produced milk is lost due to spillage and spoilage at different stages of the chain [5]. An additional concern is the decline of the consumption of fermented food due to westernization of diets [6, 7].

We reason that access to probiotic cultures for fermentation can contribute to improving health and wealth of people in resource poor countries in multiple ways: (1) increase of shelf life and microbial food safety, (2) reduce spoilage by controlled fermentations, (3) increase the nutritional properties of the fermented foods by delivery of beneficial bacteria, bioactive compounds such as vitamins, and sequestering toxic components, (4) prevent and reduce episodes of diarrhea resulting from the intake of a probiotic strain, and (5) provide incomes for local producers who sell the foods.

The ability to propagate probiotics in fermented foods is limited due to legal and biological constraints. On one hand, ownership of intellectual property and a for-profit business model limits sales to premium products. On the other hand, probiotics originating from niches in the intestinal tract often lack the metabolic capabilities to propagate well in food matrices. In addition, the continuous propagation of intestinal isolates in a dairy environment may lead to the selection of mutants with genetic rearrangements, potentially coinciding with a reduction of their probiotic functionality [8]. Recently, we reported on the concept of “generic probiotics”, as a practical solution to create increased access to probiotics for people in resource poor countries [9]. Analogous to generic drugs, we reasoned that patent-expired probiotics are free to be used by other suppliers. We applied this concept of generic probiotics, and after isolating *L. rhamnosus* GG from a commercially available product [9] we renamed it *L. rhamnosus* yoba 2012.

The failure of the probiotic *L. rhamnosus* GG strain to grow in milk, results from its inability to degrade casein and lactose [10]. In order to enable propagation of the strain, food matrices should be enriched with either degradable sugars such as glucose, proteolytic enzymes, or partially hydrolyzed nitrogen sources such as yeast extract. All three solutions lead to higher cost and technical complexity. As an alternative low-cost solution we identified a proteolytic strain of *S. thermophilus* able to degrade casein and lactose that would allow *L. rhamnosus* yoba 2012 to propagate.

In the present work, we aimed at creating a novel bacterial consortium that can be easily distributed and used for local production of fermented foods with increased food safety and nutritional values, including a genetically stable probiotic strain. The newly formulated *S. thermophilus* C106 and *L. rhamnosus* yoba 2012 consortium was shown to be sufficiently stable. Under the applied conditions, the probiotic strain *L. rhamnosus* yoba 2012 appeared to retain its pili, which are important for adhesion to the intestinal epithelium and related health effects [11–13].

## Results

### Isolation, characterization and genome sequence of *S. thermophilus* C106

*Streptococcus thermophilus* C106 was isolated from an artisanal cheese produced in Ireland, and classified on the basis of its 16S rRNA sequence (data not shown). The draft genome sequence of *S. thermophilus* C106 has been deposited at GenBank, BioProject ID PRJNA288538. The full genome sequence with a GC content of 39.0 %, comprises 1.77 Mbp assembled into 87 contigs and a total number of 1416 functional proteins have been annotated. The predicted functions of the proteins in COG categories [14] showed a rather equal distribution into cellular processes and signalling (18 %), information storage and processing (25 %) and metabolism (37 %) functions. Approximately 20 % of the predicted proteins have not been assigned a specific function (Additional file 1: Figure S1).

**Fig. 1:**
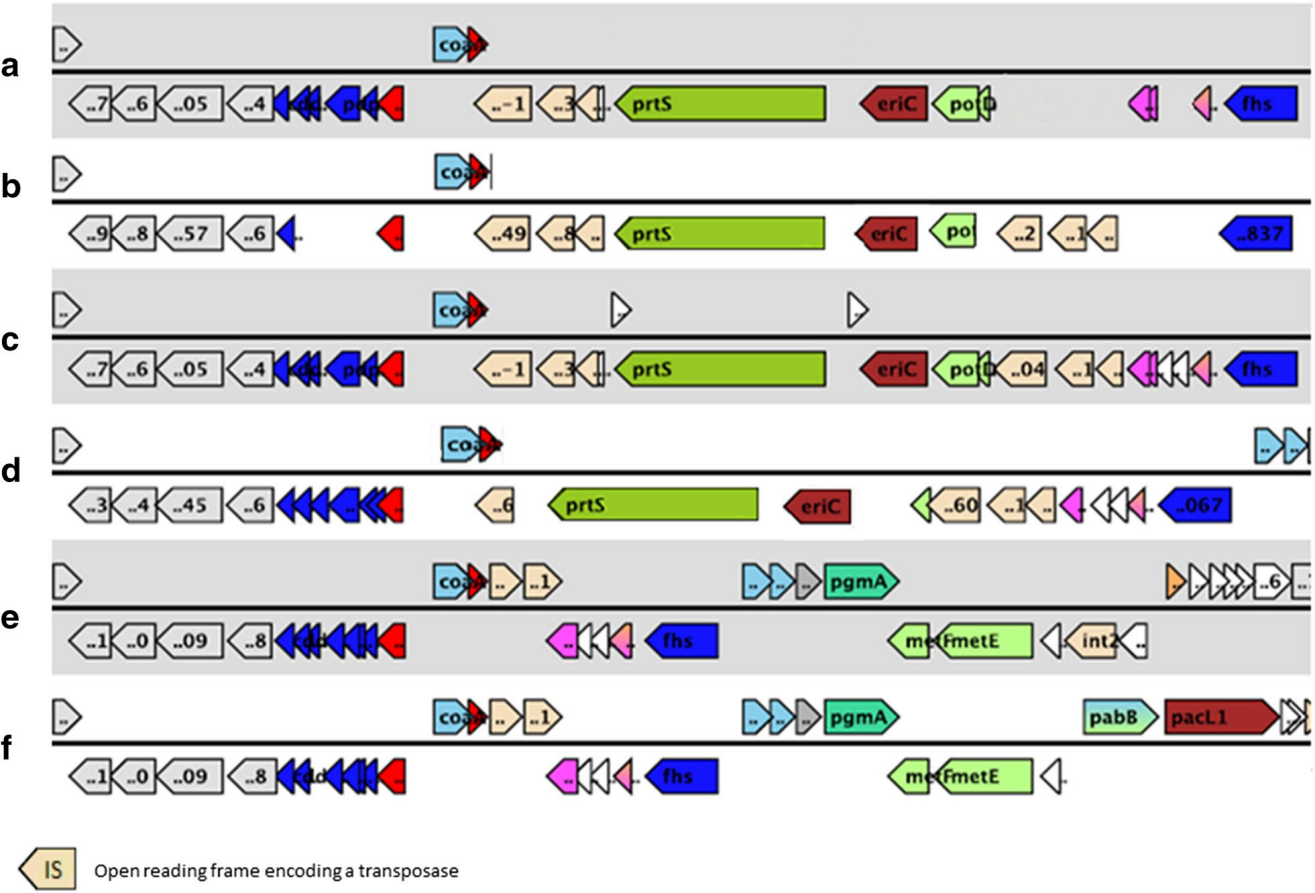
Chromosomal regions of different streptococci genome sequences. **a** *S. thermophilus* C106; **b** *S. thermophilus* LMD-9; **c** *S. thermophilus* JIM8232; **d** *S. thermophilus* MN-ZLW-002; **e** *S. thermophilus* CNRZ1066 and **f** *S. thermophilus* LMG 1831 depicting the genomic island flanked by mobile elements of four open reading frames: *potC* (truncated), *potD*, *eriC*, *prtS*. Other genes in this figure are *coaA* (pantothenate kinase), *ciaH* (sensor protein), *fhs* (formate-tetrahydrofolate), *pgmA* (phosphoglucomutase), *metF* (methylenetetrahydrofolate reductase), *metE* (5-methyltetrahyropteroltriglutamate-homocysteine S-methyl transferase), *pabB* (para-amino benzoate synthase component I), *pacL1* (Ca^2+^, Mn^2+^, P-ATPase)

The annotated genome sequence confirms the ability of *S. thermophilus* C106 strain to grow in milk by its endogenous metabolic capacity. A functional annotation of the genome reveals the presence of genes required for lactose catabolism (*lacAYZ*) operon, casein degradation (*prtS*) and transporters for peptides and dipeptides (*oppABCDF*, *dtpABCDF*). The genomic organization of the cell wall proteinase encoding gene (*prtS)* in *S. thermophilus* C106 is comparable to the one reported by Delorme et al. for *S. thermophilus* LM9-D and JIM8232 [15]. In the latter strains the proteinase gene is located in a 15 kb intergenic genomic island, between the pseudo gene *ciaH* and the gene *rpst*, as indicated in Fig. 1. Different from the findings of Delorme et al. [15], we were not able to confirm the presence—in silico—of mobile elements on both flanking regions of the *prtS* gene in *S. thermophilus* C106, but only in the upstream region. This is likely because mobile elements are repetitive sequences easily missed during the assembly and annotation process.

In addition, the annotated genome sequence indicates that *S. thermophilus* C106 is able to synthesize all essential amino acids, including histidine, methionine and glutamate, which is considered to be an indispensable metabolic property for growth in milk, as glutamate and methionine are scarce in milk with concentrations of approximately 45 mg and <1 mg l^−1^ [16], which are below the minimally required levels of 200 and 60 mg l^−1^, respectively [17]. It should be noted that auxotrophy for histidine and methionine is common among streptococci, see e.g. *S. thermophilus* LMG18311 [18].

**Fig. 2:**
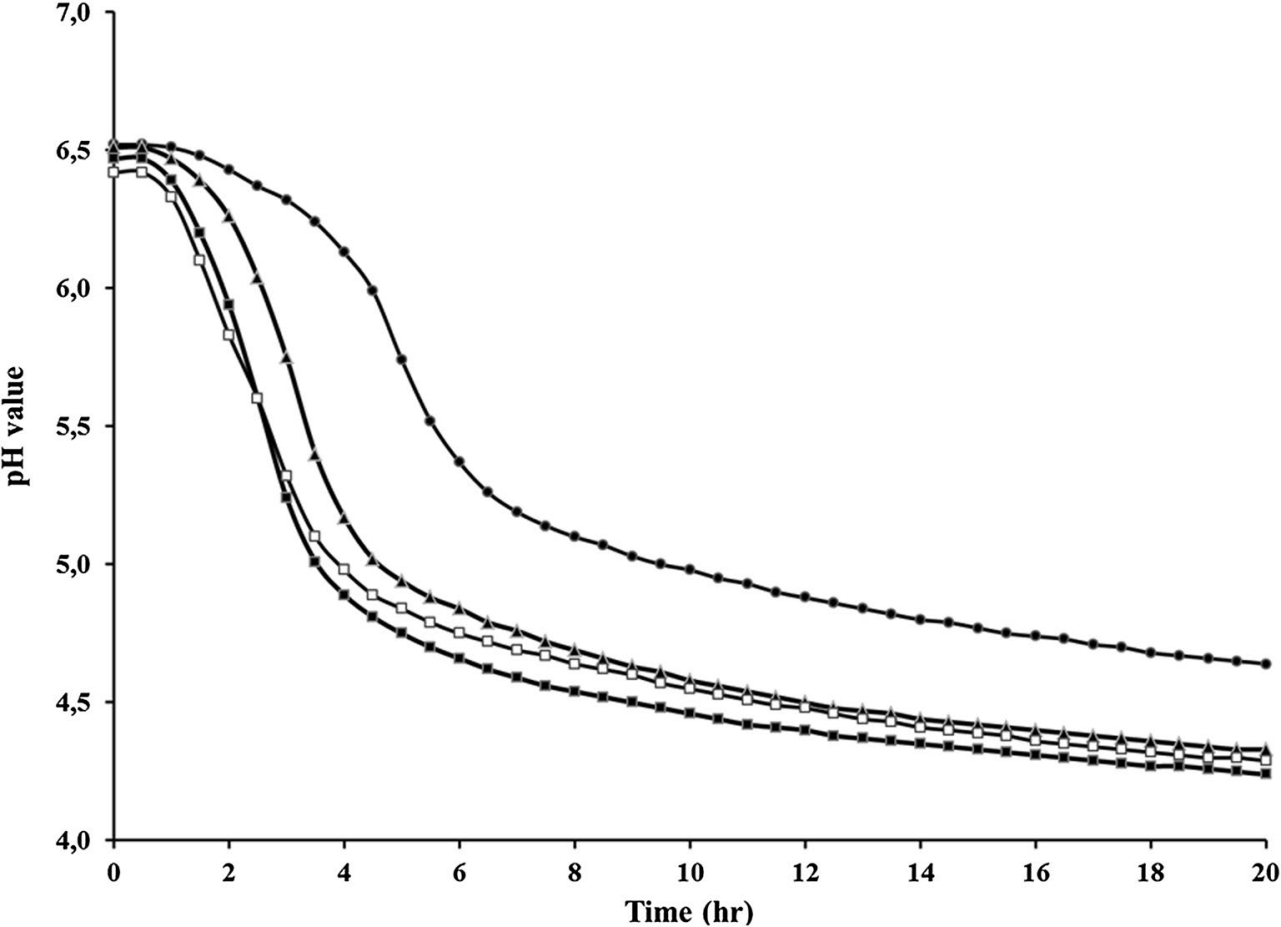
Fermentation profile of *S. thermophilus* C106 in milk at different temperatures. (*filled circle*) 32 °C; (*filled triangle*) 37 °C; (*filled square*) 42 °C; (*unfilled square*) 45 °C

The growth of *S. thermophilus* C106 in milk as sole carbon and nitrogen source was measured at temperatures of 32, 37, 42 and 45 °C (Fig. 2). Above 32 °C the strain was able to produce enough lactate to acidify the milk from pH 6.2 until pH 4.2 within 24 h. The production of lactate (as expressed in the difference in pH as a function of time) by *S. thermophilus* C106 increased at higher temperatures, reinforcing the thermophilic character of *S. thermophilus* C106. The highest delta pH/time (0.80), and thus the optimal growth temperature was found at 42 °C. Cell count analysis at t = 0 h and t = 24 h, at the optimal temperature, indicated that the propagation in milk resulted in a 4 log CFU increase during this cultivation time.

### Metabolic interactions between *S. thermophilus* C106 and *L. rhamnosus* GG

A combined analysis of the genomes of *S. thermophilus* C106 and *L. rhamnosus* GG [12, 19], and its generic variant *Lactobacillus rhamnosus* yoba 2012 used for this study [8], revealed potential interactions between the *Streptococcus* and *Lactobacillus* strains during growth in milk (Table 1). The interactions were compared to those of the classical yoghurt consortium *S. thermophilus* and *L. bulgaricus* [20], and references therein. While *L. bulgaricus* is proteolytic, *S. thermophilus* is usually non-proteolytic and therefore growth of most *S. thermophilus* strains can be highly improved when co-cultured with *L. bulgaricus,* as they profit from released peptides and amino acids. *L. bulgaricus* lacks a pyruvate-formate lyase and might be supplied with formate by *S. thermophilus* as well as with folic acid, which is required for purine and amino acid biosynthesis. Furthermore, *L. bulgaricus* benefits from the carbon dioxide released by *S. thermophilus,* which is used for amino acid and nucleotide biosynthesis. Since *L. bulgaricus* misses parts of the biosynthetic pathway needed for the biosynthesis of long chain unsaturated fatty acids, which can promote growth, one can speculate that *S. thermophilus* also would also supply long chain unsaturated fatty acids to *L. bulgaricus*. It is further suggested that both organisms exchange ornithine and putrescine in order to increase their resistance to oxidative stress.

**Table 1:** Potential interactions between *S. thermophilus* and *Lactobacillus species*

Classical yoghurt consortium			Novel consortium		
Compound	*S. thermophilus*	*L. bulgaricus*	Compound	*S. thermophilus* C106	*L. rhamnosus* yoba 2012
Amino acids	Consumes	Provides	Folic acid	Provides	Consumes
Carbon dioxide	Provides	Consumes	Galactose	Provides	Consumes
Fatty acids	Provides	Consumes	Glycerol	Provides	Consumes
Folic acid	Provides	Consumes	Peptides	Provides	Consumes
Formic acid	Provides	Consumes	Succinate	Provides	Consumes
Ornithine	Provides	Consumes	Xanthine/Guanine	Provides	Consumes
Peptides	Consumes	Provides			
Putrescine	Consumes	Provides			
Pyruvatic acid	Provides	Consumes			

Metabolic interactions include those in the classical yoghurt consortium of *L. bulgaricus* and *S. thermophilus* and between *S.thermophilus* C106 and *Lactobacillus rhamnosus* yoba 2012

The potential interactions between *S. thermophilus* C106 and *L. rhamnosus* yoba 2012 were determined by using genome-scale models (see Methods for details). The models suggest that *L. rhamnosus* yoba 2012 is not capable of producing folic acid, which therefore needs to be provided by C106 (Table 1). Moreover, C106 needs to provide either xanthine or guanine to *L. rhamnosus* yoba 2012, both of which are involved in purine metabolism. We also observed an excretion of succinate and glycerol by C106, which can both be metabolized by *L. rhamnosus* yoba 2012. The *L. rhamnosus* strain cannot grow on lactose, however on galactose [21], which is released by C106, as concluded from HPLC analysis of milk fermented by the consortium (data not shown). While this interaction is revealed by our stoichiometric model, the uptake of galactose by *L. rhamnosus* yoba 2012 may not be essential for growth since in silico it could also utilize succinate or glycerol made by C106 as described above. In the experimental set-up, however, an accumulation of galactose in the media would be disadvantageous for C106 because of product inhibition. Hence, the removal of galactose by *L. rhamnosus* yoba 2012 might be beneficial for the C106 strain.

### Preparation, fermenting capacity and technical properties of *L. rhamnosus* yoba 2012 and *S. thermophilus* C106 seed culture

**Fig. 3:**
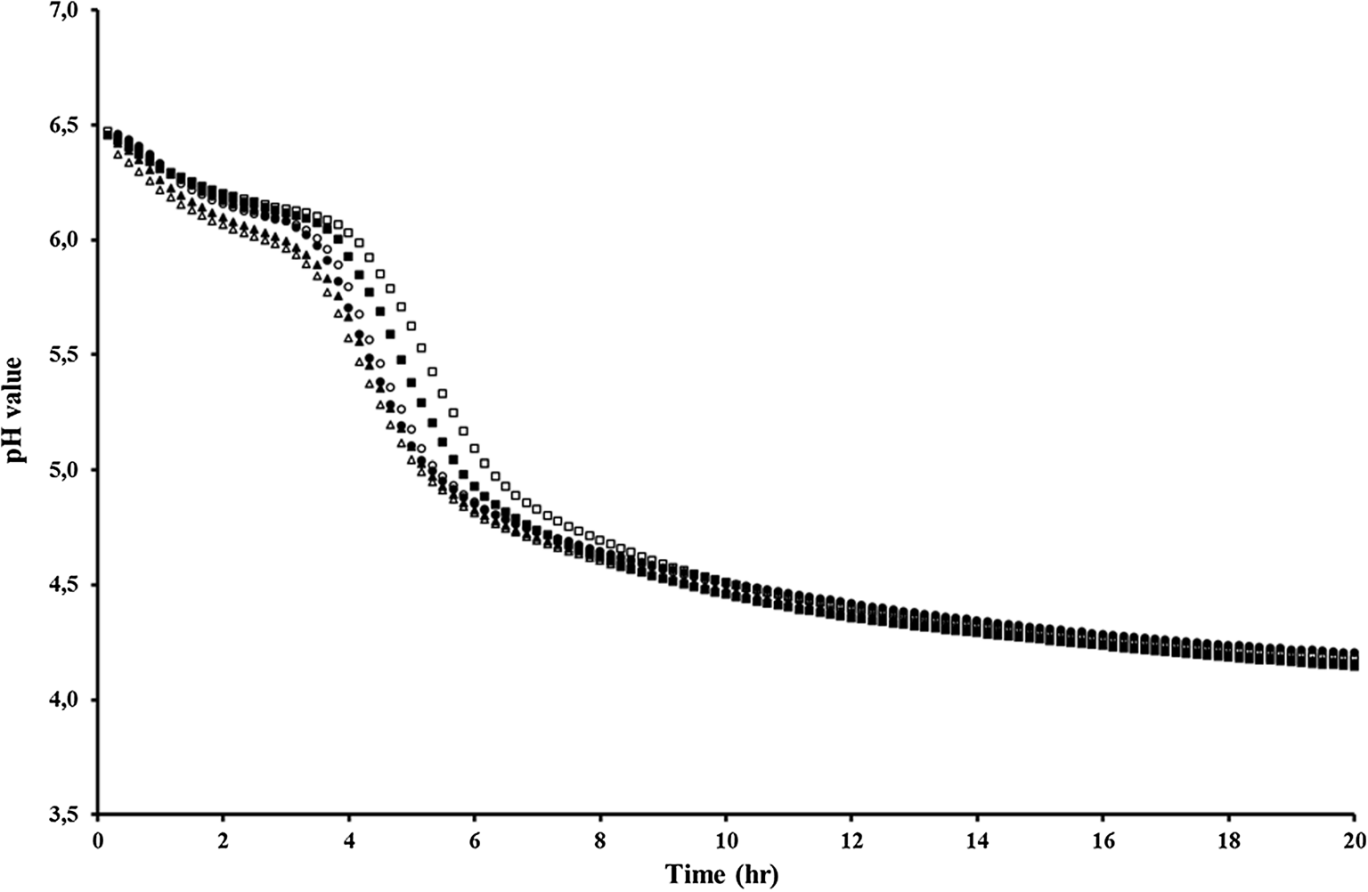
Fermentation capacity of seed cultures in pasteurized semi-skimmed milk inoculated after different storage time and at different growth temperatures. 45 °C, 2 years old (*filled circle*, *unfilled circle*); 45 °C fresh (*filled triangle*, *unfilled triangle*); 37 °C 2 years old (*filled square*, *unfilled square*)

The production process for the dried seed cultures and its subsequent application for the preparation of fermented foods are depicted in Table 2 and Additional file 2: Figure S2. In a typical experiment, one gram of seed culture containing *L. rhamnosus* yoba 2012 and *S. thermophilus* C106 in concentrations of 5E+09 CFU g^−1^ each, was used as inoculum for one l of semi-skimmed milk. Growth temperature can vary between 37 and 45 °C. After a short lag phase, the milk acidified until pH 4.3, while both the *L. rhamnosus* and *S. thermophilus* strains propagated and reached titers of 7.8E+08 and 1E+09 CFU ml^−1^, respectively (Table 3, rows 3 and 4). The fermentation profile (pH decrease as a function of time) is depicted in Fig. 3. For the so-called main fermentation, 1 % (v/v) of fermented milk produced by the first round of fermentation was used as an inoculum. The final pH and cell titers were similar to those observed after the first fermentation (Table 3 rows 4 and 5). In addition to pH and CFU analyses, HPLC analyses indicated a consumption of lactose and production of galactose and lactic acid (data not shown).

**Table 2:**
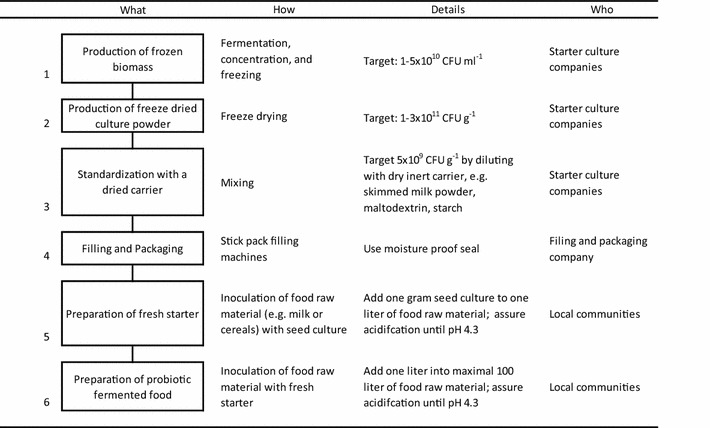
Seed culture production and application protocol

In order to assure food safety of the produced steed cultures, the seed cultures were checked for absence of pathogen and spoilage organisms after step 4

**Table 3:** Propagation of *Lactobacillus rhamnosus* yoba 2012 in a variety of fermented foods

Product	Origin/site	Raw materials/Composition	After fermentation (CFU/ml)					
			*L. rhamnosus*	*S. thermophilus*	*S. th/L.rha*	t (h)	T (°C)	pH
Yoba	Uganda	Semi-skimmed milk^a,d^	2.1E+07	7.8E+08	37	16	45	4.3
Yoba	Uganda	Semi-skimmed milk^a,d^	1.8E+07	9.0E+08	50	16	45	4.3
Yoba	Uganda	Semi-skimmed milk^b,d^	1.3E+07	1.60E+09	123	16	45	4.3
Yoba	Uganda	Semi-skimmed milk^b,d^	9.5E+06	1.90E+09	200	16	45	4.3
Yoba	Kenya	1000 ml of milk^c^	4.0E+07	ND	–	16	45	ND
Yoba	Kenya	1000 ml of milk^c^	2.2E+07	ND	–	16	45	ND
Yoba	Uganda	1000 ml of milk^c^	6.2E+07	1.6E+09	26	16	37	4.4
Yoba	Uganda	1000 ml of milk^c^	7.9E+07	1.5E+09	19	16	37	4.4
Yoba	Uganda	1000 ml of milk^b^	6.5E+07	2.4E+09	37	16	37	4.3
Yoba	Uganda	1000 ml of milk^b^	5.6E+07	2.3E+09	41	16	37	4.3
Yoba	Uganda	1000 ml of milk^c,d^	2.6E+07	5.1E+08	20	16	45	4.4
Yoba	Uganda	1000 ml of milk^c,d^	3.5E+07	8.0E+08	23	16	45	4.4
Yoba	Uganda	1000 ml of milk^b,d^	1.7E+07	1.7E+09	100	16	45	4.3
Yoba	Uganda	1000 ml of milk^b,d^	1.6E+07	8.0E+08	50	16	45	4.3
Zomkom	Burkina Faso	100 g wheat, 1000 ml water^b^	3.4E+08	3.3E+06	0.01	15	37	<4.3
Zomkom	Burkina Faso	75 g wheat, 250 ml milk, 750 ml water^b^	2.5E+08	5.7E+08	2	15	37	<4.3
Zomkom	Burkina Faso	50 g wheat, 500 ml milk, 500 ml water^b^	6.6E+08	2.7E+09	4	15	37	<4.3
Zomkom	Burkina Faso	25 g wheat, 750 ml milk, 250 ml water^b^	2.6E+08	2.8E+09	11	15	37	<4.3
Zomkom	Burkina Faso	0 g wheat, 1000 ml milk, 0 ml water^b^	3.8E+07	2.4E+09	63	15	37	<4.3
Obushera	Uganda	50 g sorghum in 400 ml water^c^	2.8E+08	ND	–	24	25	4.0
Obushera	Uganda	50 g sorghum in 400 ml water^c^	1.3E+08	ND	–	24	25	4.1
Uji	Kenya	Maize^b,e^	8.9E+09	ND	–	16	45	ND
Uji	Kenya	Sorghum^b,e^	7.5E+09	ND	–	16	45	ND
Uji	Kenya	Sorghum and maize (1:1)^b,e^	3.7E+09	ND	–	16	45	ND
Mutandabota	Zimbabwe	Pulp of the baobab fruit and milk^f^	6.3E+08	0	–	24	30	3.5

Fermentations were carried out at 37 °C unless stated otherwise. The cell count of *L. rhmanosu*s GG and *S. thermophilus* C106 after inoculation varied between 1E+06 and 1E+07 CFU ml^−1^

*ND* not determined

^a^First passage, one gram seed culture used as inoculum

^b^Second passage, freshly prepared liquid starter used as inoculum

^c^Two year old seed culture

^d^Incubation at 45 °C

^e^Average of three independent CFU counts per product over a period of 2 weeks

^f^Starter with exclusively *L. rhamnosus* yoba 2012 as reported by Mpofu et al. [40]

In order to test the stability and fermentation activity of the seed cultures over time, the growth experiments were repeated 2 years later using seed cultures from the same production batch stored at −20 °C. Table 4 shows the cell concentration of the two strains determined in 8 different seed cultures, and indicates a non-significant loss of viability for *S. thermophilus* (from 2.5 to 2.3E+09 CFU g^−1^) and a minor decrease in the average cell count of *L. rhamnosus* yoba 2012 (from 4.9 to 1.5E+09 CFU g^−1^). The latter decrease is acceptable as it does not significantly affect the fermentation profiles and final CFU’s of *L. rhamnosus* strain in fermented food products. Accordingly, the fermentation profile shown in Fig. 3 and the measured pH and cell count in Table 3 (rows 13–16) confirmed that the cultures had kept their fermentation capacity at 37 and 45 °C.

**Table 4:** Stability and bacterial content variability of dried seed cultures

Sachet #	2013		2015	
	*L. rha* (CFU g^−1^)	*S. the* (CFU g^−1^)	*L. rha* (CFU g^−1^)	*S. the* (CFU g^−1^)
1	4.0E+09	2.7E+09	1.4E+09	2.0E+09
2	3.1E+09	1.4E+09	1.5E+09	2.7E+09
3	6.3E+09	3.0E+09	1.4E+09	2.2E+09
4	3.9E+09	2.0E+09	1.4E+09	2.2E+09
5	3.9E+09	2.2E+09	1.2E+09	1.8E+09
6	7.7E+09	2.9E+09	1.5E+09	2.5E+09
7	5.5E+09	2.9E+09	1.1E+09	2.1E+09
8	5.1E+09	2.7E+09	2.2E+09	3.0E+09
Average	4.9 ± 1.5E+09	2.5 ± 0.6E+09	1.5 ± 0.3E+09	2.3 ± 0.4E+09

The values indicate the CFU ml^−1^ of *Lactobacillus rhamnosus* yoba 2012 (*L. rha*) and *Streptoccoccus thermophilus* C106 (*S. the*) produced on 18/01/2013 and tested on 12/07/2013 and 13/02/2015

### Robustness of piliation of *L. rhamnosus* yoba 2012 during milk fermentation

**Fig. 4:**
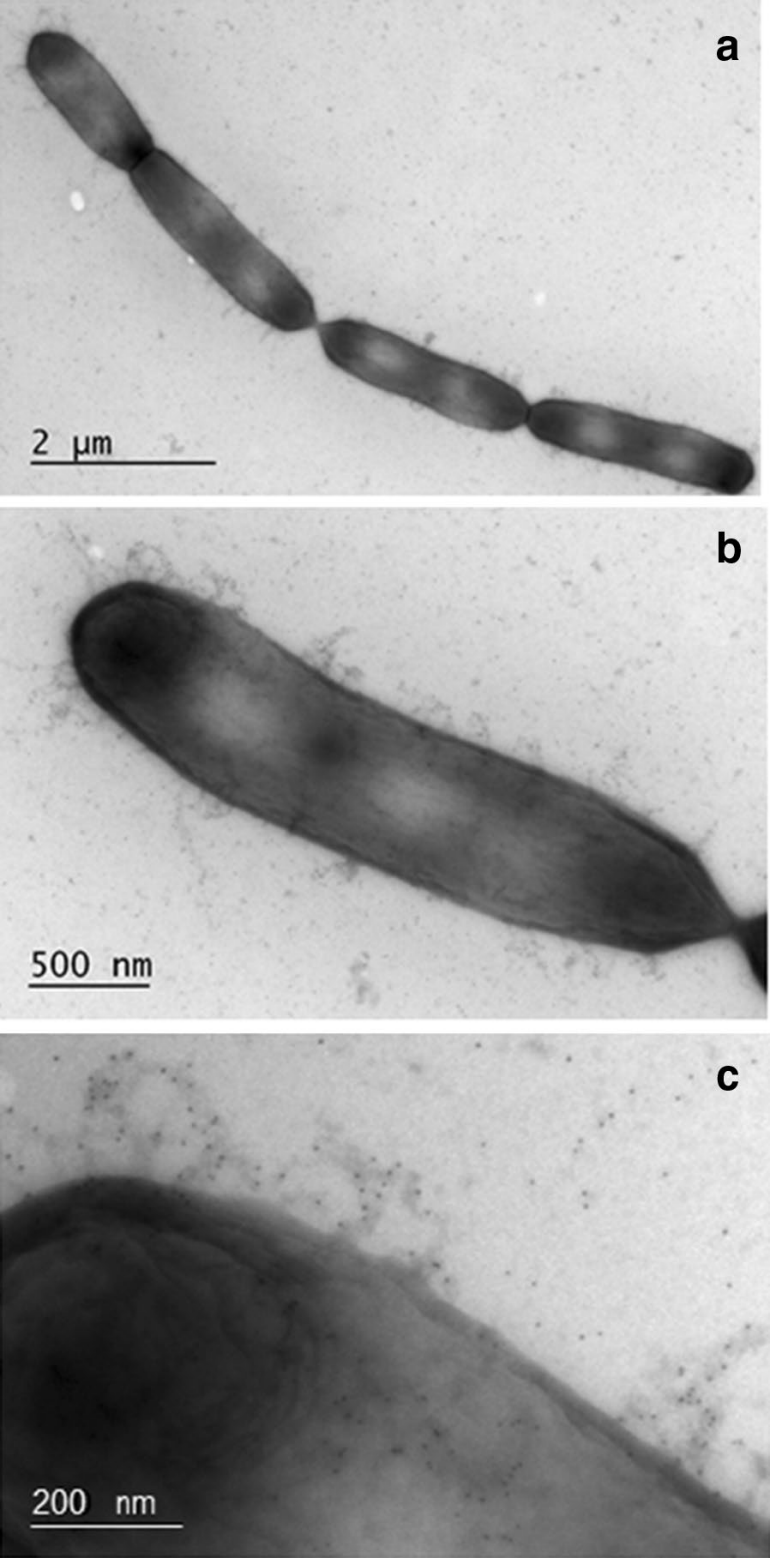
Transmission electron microscopy observations of piliated *Lactobacillus rhamnosus* strain yoba 2012. Cells were isolated from fermented milk and immuno-gold labeled with anti-SpaA antibodies and gold particule-conjugated protein A. Gold particules (5 nm) co-localize with pilus structures (*black dots* on pictures)

**Fig. 5:**
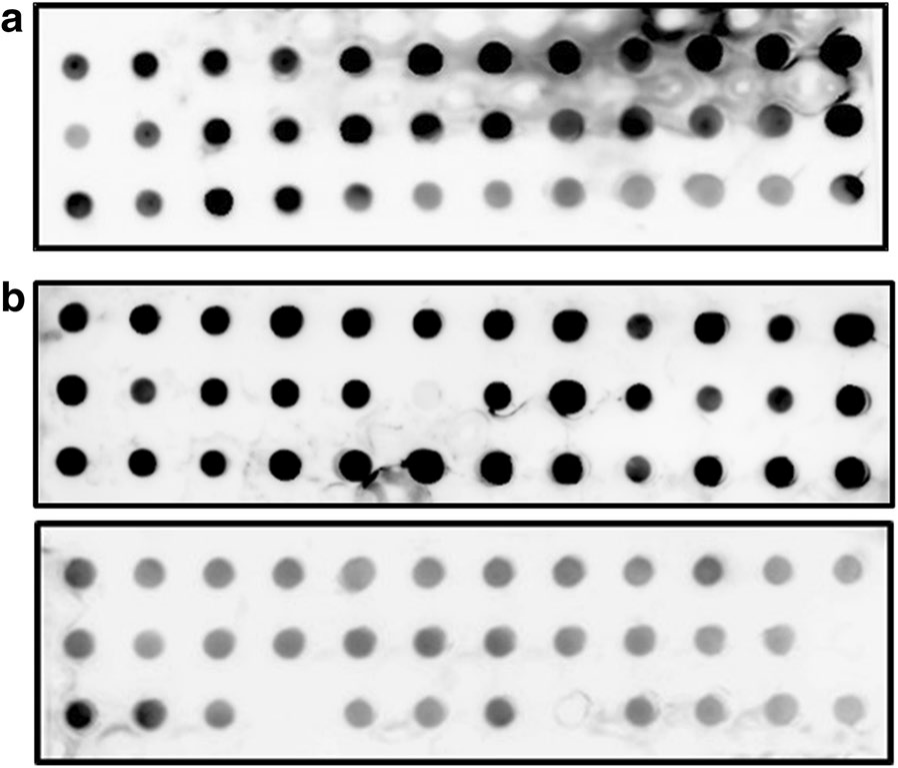
Immunoblotting analysis of fermented milk after first **(a)** and second passage **(b)** using anti-SpaA serum. Single *L. rhamnosus* colonies were randomly picked from 2-day old MRS agar plates and analyzed by immunoblotting using anti-SpaA polyclonal antibodies. In **a** 70 out of 72 colonies tested were pilus positive (97.2 %). In **b** 101 out of 108 colonies tested were pilus positive (93.5 %). Note: a third immunoblotting (not shown in Fig. 5) was performed for sample 2b and included in the piliation percentage calculation above. Piliation phenotype of some colonies tested was further confirmed by PCR analysis as described in the “Methods”

Previously, we reported that the genome of *L. rhamnosus* GG is prone to genetic rearrangements, especially around the insertion sequence flanked genomic islands containing the *spa*CBA-*srt*C1 pili encoding genes [8]. Therefore, we checked for the presence of pili after the first and second fermentation of the milk. The generic variant of *L. rhamnosus* GG used in this study, *L. rhamnosus* yoba 2012, harbored pilus structures, as observed by immunogold-staining TEM (Fig. 4). Immunoblotting with anti-spaA antiserum on 36 *L. rhamnosus* colonies isolated from the first passage were all piliated, whereas immunoblotting on 72 *L. rhamnosus* colonies isolated after the second passage indicated that 4 colonies were pilus negative (Fig. 5). The observed pilus phenotypes were confirmed by LGG-specific and pilus-specific (the *srtC1* gene) PCRs for a selected number of colonies. Quantitative PCRs performed on fermented milk samples after the first and the second passage did not show a detectable loss of pilus-specific genes (data not shown), confirming that the loss of these genes was a marginal in terms of its impact on probiotic functionality.

### Application of *L. rhamnosus* yoba 2012 and *S. thermophilus* C106 seed culture on other food raw materials

In order to further investigate the potential of using *L. rhamnosus* yoba 2012 and *S. thermophilus* C106 seed cultures for preparation other traditionally fermented foods, we tested production of zomkom (Burkina Faso, wheat and milk), obushera (Uganda, sorghum), uji (Kenya, maize or sorghum) and mutandabota (Zimbabwe, fruits of the baobab tree and milk). Table 3 shows that all ingredients were well-fermented down to a pH of approximately 4.3. Depending on the specific food matrix, the titer of *L. rhamnosus* yoba 2012 and *S. thermophilus* C106 increased up to a factor of 1000 resulting in a final titer of >1E10^9^ CFU ml^−1^. Furthermore, *L. rhamnosus* propagated well in wheat, sorghum and maize. In contrast, *S. thermophilus* did not multiply without the presence of milk in wheat, despite the growth of *L. rhamnosus* in this food matrix.

### Food safety and sensory evaluation

In addition to the technical evaluation, the fermented products were subjected to food safety tests, as required for human consumption, prior to sensory evaluation by local Ugandan panel members. In a total of 10 sachets each containing 1 g of seed culture, the following results were obtained: no detectable levels of Enterobacteriaceae, less than 100 CFU g^−1^ of non-lactic acid bacteria, less than 10 CFU g^−1^ of yeasts and moulds, no detectable levels of *Listeria monocytogenes* and no detectable levels of *Salmonella*.

Typical sensory aspects such as texture, viscosity, creaminess, taste, sourness, sweetness and mouthfeel were evaluated. Whitening ability and whey separation were also evaluated for the fermented milk products. Despite the large variation of the scores, likely as a results of different raw materials and not always perfectly controlled processing conditions in the African setting, the overall liking and general acceptability of all fermented products were positive (data not shown).

## Discussion

Africa has an extensive history of production and a rich variety of traditionally fermented foods. These type of foods have a large impact on the nutrition, health and socio-economy of the people of a continent that is often challenged by war, drought, famine and disease. So far the use of indigenous foods as potential vehicles for delivering beneficial bacteria received little attention, notwithstanding their great potential for prophylactic and therapeutic use in resource poor countries [4].

Here we describe the development and the characterization of a stable dried seed culture that induces the propagation of probiotic *L. rhamnosus* yoba 2012 in milk, fruit and cereal-based food matrices. Through the addition of *S.* *thermophilus* C106 to the seed culture, we were able to overcome the metabolic limitations of *L.* *rhamnosus* to grow in milk, a widely-available raw material in rural regions of sub-saharan Africa: Degradation of casein and lactose was carried out by *S.* *thermophilus,* which provided peptides and monosaccharides, including galactose, that could be subsequently be used by the *L.* *rhamnosus* strain.

In traditional yoghurt fermentations, *S. thermophilus* stimulates *L. bulgaricus* via lipolysis and by supplying formic acid, folic acid and CO_2_, which are all involved in purine metabolism. In turn, *L. bulgaricus* provides *S. thermophilus* with peptides and amino acids released from milk casein by its exoprotease PrtB. Exopolysaccharides are reported to potentially facilitate these nutritional exchanges [22]. Contrary to the proto-cooperation of the usual yoghurt strains, the *S.* *thermophilus* C106 is able to grow in milk without the metabolic activity of a *Lactobacillus* strain. Although the removal of galactose by *L. rhamnosus* yoba 2012 might increase the final titres of *S. thermophilus* C106, detailed evidence for mutual metabolic dependencies and benefits for both the *S.* *thermophilus* C106 and *L. rhamnosus* yoba 2012 consortium should be gained from longitudinal transcriptome analysis during fermentation [22].

The use of two strains provides an affordable way of delivering dried seed cultures at an affordable price for people living in resource poor settings. To reach 100 l production, the strains are first grown in 1 l followed by passage to the larger milk volume (Table 3; Fig. 3). Multiple analyses of the final titre of *L. rhamnosus* yoba 2012 in the fermented milk indicated a concentration of approximately 8E+07 CFU ml^−1^, which would result in a daily dose of at least 1E+10 CFU upon consumption of 200–250 ml of the fermented drink.

**Fig. 6:**
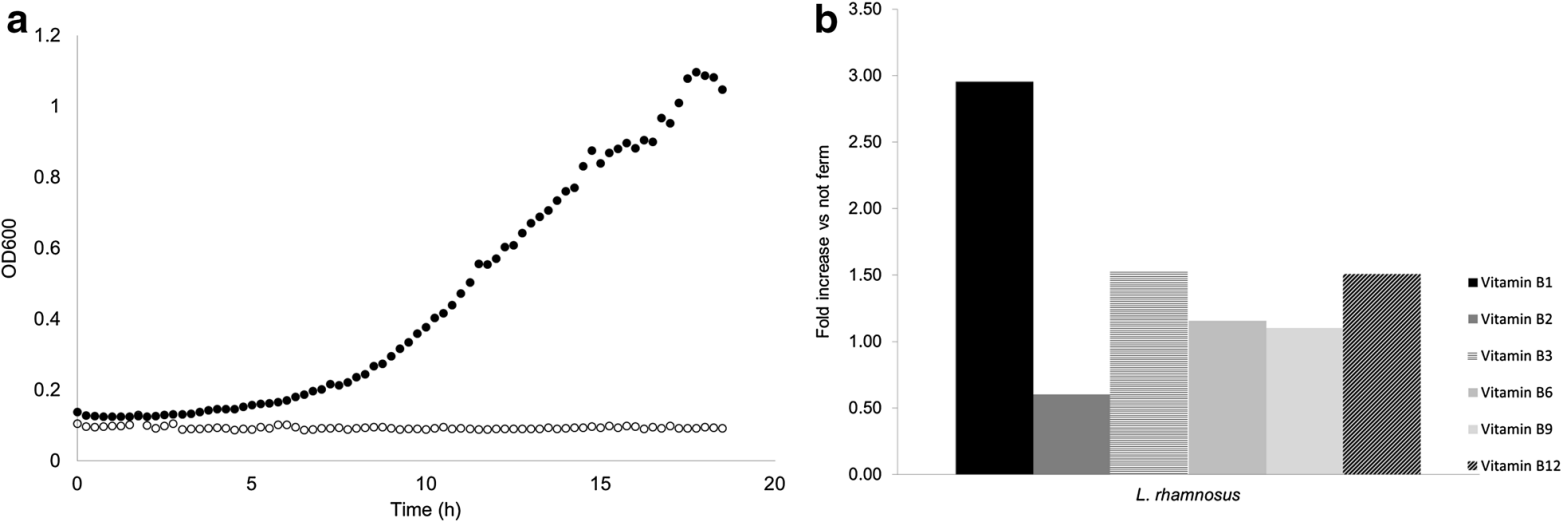
Additional benefits of foods fermented with *Lactobacillus rhamnosus*. **a** Growth of the pathogen *Cronobacter sakazakii* in sorghum in presence (*unfilled circle*) and absence (*filled circle*) of fermenting *Lactobacillus rhamnosus*. **b** Change in vitamin B content of soy after and before fermentation

A well-accepted aspect of fermented food is its food preservation capacity and increased nutritional value compared to the non-fermented raw material. The use of the present combination of lactic acid bacteria will help to avoid spoilage, enhance food safety and increase the nutritional value by delivery of vitamins [23]. Examples of such characteristics are shown by the prevention of outgrowth of the food pathogen *Cronobacter sakazakii* in a sorghum matrix as a result of the acidification by lactic acid produced by *L. rhamnosus* GG and potentially other antagonistic effects induced by specific antimicrobial peptides (Fig. 6a). This finding of enhanced microbial food safety is further supported in a recent study indicating the efficient suppression of five food pathogens in an African dairy product fermented with *Lactobacillus rhamnosus* yoba 2012 [24]. In addition, we evaluated the change in concentration of B vitamins as a result of the fermentation process. We observed a 3-fold increase of vitamin B1 or thiamine, while the other B vitamins remained at similar concentrations to those found prior to fermentation (Fig. 6b).

In addition, detoxification is a well-accepted benefit of some food fermentations [25]. Although not tested here, *L. rhamnosus* GG has been shown to bind and neutralize toxins known to contaminate foods, leading to a reduction of their uptake in the gastro-intestinal tract as they are secreted with *L rhamnosus* GG during defecation. [26, 27]. In addition, probiotic *L. rhamnosus* GR-1 supplemented yogurt has been shown to lower mercury and arsenic uptake in children and pregnant women [28].

Another probiotic functionality of *L. rhamnosus* GG relates to adhesion. Recent studies indicated that the pilus encoding *spaCBA*-*srtC1* genes are responsible for epithelial adhesion and subsequent induction of cellular responses [11, 13]. In the present consortium and its subsequent use for yoghurt production, we could show that only minor depiliation was observed with the *L. rhamnosus* strain. On this basis, we conclude that the new fermented milk product described here did not significantly lose probiotic activity related to adherence capacity during two passages in the yoghurt production process.

In a more clinical context, a number of studies have shown that oral administration of *L. rhamnosus* GG bacteria results in in a reduction of the duration of diarrhoea [29]. Meta-analyses of clinical studies led to scientific consensus for *Lactobacillus rhamnosus* GG as a probiotic that upon oral intake is able to consistently shorten the diarrheal phase of rotavirus infection by one day with no reported adverse events, see e.g. [30, 31]. Notably, this diarrheal disease is a major concern among children in sub-Saharan Africa, including Uganda [32]. We plan to further substantiate the health benefits of affordable generic probiotics by intervention studies among local communities in Uganda. The outcome of these studies could provide more insights in specific health benefits brought by fermented foods produced by controlled fermentation, including so-called locally sourced probiotics [33], in addition to the generic use of a globally sourced probiotics as described here.

It should be noted that the current practices of traditional fermentation are mostly restricted to the house-hold level and are not suitable for commercialization as a result of poor and inconsistent quality of final products. The distribution of the Yoba seed cultures allows small entrepreneurs and cooperatives to produce fermented food in a controlled manner, which renders these products suitable for consumer acceptance and local commercialisation. Apart from the improved quality, the use of seed cultures for controlled fermentation prevents spoilage and results in reduced food losses. According to our observations, the introduction of the seed cultures in rural Uganda does not affect traditional fermented food practices at the house-hold level. People who rely on rural homesteads for their meals including traditional fermented foods will continue to do so, since this often concerns own farm produce whereby no costs are involved. However, people who have budget to take food in villages and trading centres tend to switch to yoghurt instead of other, often unhealthy and less nutritious types of food and soft drinks. To date, seed cultures containing the two strains described in this paper have been implemented by 46 dairy cooperatives and local producers in Uganda, resulting in sales of over 8000 l of probiotic fermented milk per week in mostly rural areas. This has benefitted over 25,000 people, including producers and consumers (Additional file 3: Figure S3) with the potential to reach hundreds of thousands in the near future.

## Conclusions

We have described the creation of a novel probiotic formulation of two lactic acid bacteria that is affordable and practical for use in resource-challenged communities in Africa. An essential feature of the formulation is strain *S. thermophilus* C106 which complements the disability of *L. rhamnosus* yoba 2012 to grow in milk by degrading casein and lactose. The entire production process has been validated and is carried out in rural areas and only requires the sachet, milk, a sauce pan and a source of heat. The freeze-dried strains stored in moisture-proof sachets remain active over a period of at least 2 years. This initiative provides a means to bring highly nutritious, health-promoting food to people around the world who currently have no access to probiotic benefits.

## Methods

### Genome-scale metabolic model of *L. rhamnosus* yoba 2012 and *S. thermophilus* C106

Genome scale models for *S. thermophilus* C106 and *L. rhamnosus* yoba 2012 have been created based on the NCBI reference sequences NC_013198.1 and NC_006448.1, respectively, using FiJo: in-house developed software based on the Autograph method [34] using *L. plantarum* WCFS1 and *S. thermophilus* LMG18311 as reference models. Non-gene associated reactions from *L. plantarum* and *S. thermophilus* were added to the models of *L. rhamnosus* yoba 2012 and *S. thermophilus* C106, respectively. Details in cell wall components and fatty acid biosynthesis were copied from the reference models as they were not considered to affect the metabolic interactions and would require more detailed scrutiny outside of the scope of this paper. Potential interactions have been revealed by forcing equal growth rate to both organisms which would be required for balanced growth of the consortium [35] and subsequently disabling transport reactions for shared metabolites. Through flux balance analysis (FBA) with growth rate as objective function, we scored which reactions abolished growth. The media consists of biotine, lactose, milk peptides, nicotinate, pantothenate, phosphate, pyridoxamine, riboflavin, thiamin and water. All simulations have been performed using CBMPy associated to PySCeS [36].

### Preparation of the dried seed culture

The strain *Lactobacillus rhamnosus* yoba 2012 [9]; strain LMG 27229 was isolated from a powder dairy formula by Sybesma et al. [8]. The *Streptococcus thermophilus* C106 strain (CSK Food Enrichment culture collection, Ede, The Netherlands) was isolated from an artisanal cheese in Ireland. LMG 27229 and *S. thermophilus* C106 were cultivated in 100 and 12,000 l batch fermentations, respectively, harvested by centrifugation, supplemented with lactose as a cryoprotectant, frozen in pellets using liquid nitrogen and stored at −55 °C, following CSK food enrichment fermentation recipes.

The cell concentration of *L. rhamnosus* yoba 2012 and *S. thermophilus* C106 in the frozen pellets was determined by using plate assays of MRS (Biotrading, article K511F200GV) or M17 (Biotrading, K464F200GV) supplemented with 0.5 % lactose, respectively. The frozen pellets were also checked for the presence of *Enterobacteriaceae* (absent in 10 ml), non-lactic acid bacteria (<100 CFU ml^−1^), yeasts and moulds (<10 CFU ml^−1^), *Listeria monocytogenes* (absent in 1 ml) and *Salmonella* (absent in 1 ml). Two kg of liquid frozen pellets were freeze dried (GR Instruments; Wijk bij Duurstede, Netherlands) for 48 h at −40 °C. Next, the freeze dried residue was grinded into a homogenous powder, followed by cell count analysis of the two strains. The bacterial powders were blended with an inert maltodextrin up to a target concentration between 5E+09 and 1E+10 CFU g^−1^. Finally, the bacterial powder was filled under humidity-controlled conditions in water-tight sachets with total dried powder content of one gram per sachet. The entire seed culture production process is shown in Table 2.

### Acidification profiles of seed cultures

Milk was pre-treated by a heating step of 30 min at 102 °C and aliquoted into four 250 ml glass bottles. The temperature of each bottle was adjusted to 32, 37, 42 or 45 °C using different water baths. Frozen pellets of *S. thermophilus* C106 with known starting viability were dosed (0.25 g l^−1^) into 200 ml milk. For each cultivation the pH was monitored every 30 min for a total of 240 h.

#### Immunoblotting analysis

From 48 h MRS plates, two distinct colony morphologies could be observed: large white colonies and very small white colonies, corresponding, respectively, to *L. rhamnosus* and *S. thermophilus* cells. *L. rhamnosus* colonies were randomly picked from seed culture sachets, first and second passages of fermented milk for further analysis. Expression of SpaA pilin subunits in *L. rhamnosus* cells was assessed using a immunoblotting approach where single colonies were transferred into 96-well plates and analyzed by immunoblotting using polyclonal anti-SpaA antibodies, as primary antibody and goat anti-rabbit antibodies conjugated to horse radish peroxidase, as secondary antibody (Biorad Laboratories Inc., Hercules, CA, USA). The overall method has been described previously [10].

#### Colony PCR-screening

*Lactobacillus rhamnosus* colonies were picked from MRS agar plates, re-suspended in 20 µl double distilled water and microwaved for 3 min to break down the bacteria cells. Next, 2 µl of each picked colony was added to Green Dream Taq Master Mix (Thermo Scientific) as per manufacturer’s instructions using one of the two different sets of primers, as described below. Amplicons were analyzed by 1 % (w/v) agarose gel electrophoresis. The sequences of the LGG-specific primers were based on a previous study [37], the srtC1-specific primers include the forward primer 5′-AAACCGCCCAACTTGAAGCCTC-3′ and the reverse primer 5′AAAGTTAATAAGATAAATGAG-3′.

#### Immuno-staining transmission microscopy

Samples were observed by transmission electron microscopy (JEOL 1400, Jeol Ltd., Japan), as previously described [38]. Gold particles with a diameter of 5 nm were conjugated to protein A and polyclonal anti-SpaA antibodies were employed to visualize pilus structures on the cell surface of *L. rhamnosus*.

#### Preparation of probiotic fermented foods

In a typical experiment, the content of a sachets (one gram) was used to inoculate 1 l of semi-skimmed and pasteurized milk (1.5 % fat, 3.5 % protein), and incubated at 37 or 45 °C for approximately 12 h, until pH 4.3 (first passage). The fermented milk was subsequently used as inoculum for the production of maximal 100 l fermented milk or other fermented food (second passage).

Fermentations in milk were carried out for pilot experiments in the laboratory of CSK Food Enrichment, Ede, The Netherlands as well as for yoghurt^1^ production in African countries, including Uganda, Kenya, Tanzania, Zambia, Zimbabwe, and Burkina Faso (Table 3). Obushera—a sorghum-based beverage [39]—was fermented with the Yoba seed culture in the Department of Food Technology and Nutrition, Makerere University, Kampala, Uganda for a period of 24 h at 25–27 °C.

Uji was prepared at the Jomo Kenyatta University of Agriculture and Technology in Kenya. Sorghum, maize flour or a mixture of both was added to water to a final concentration of approximately 6 % (w/v), boiled to obtain a thick porridge and cooled down to 45 °C prior to inoculation with the Yoba seed culture. Zomkom, a sorghum-based beverage from Burkina Faso, was prepared in The Netherlands by boiling one l of water with sorghum or wheat flour, full fat milk, 5 % ginger syrup (v/v) and 3 % dark brown sugar (w/v), as specified in Table 3. After pasteurization and continuous stirring, the mixture was cooled down to 43 °C and a freshly prepared fermented milk starter (1–2 %) was added. The mixture was incubated at 37 °C for 15 h.

The preparation of fermented mutandabota—a product from the fruits of the baobab tree and milk—in Zimbabwe has been reported previously [40].

Cell counts for *L. rhamnosus* and *S. thermophilus* were determined on MRS agar medium (Difco Laboratories, Detroit, MI, USA) and LM17 (Difco Laboratories), respectively, by using commonly applied microbiological plating methods.

### Food safety of seed cultures and sensory evaluation of dairy and cereal-based fermentations

The content of the seed cultures were routinely tested by selective enumeration methods: *Listeria monocytogenes* according to ISO 11290-2, Enterobacteriaceae yeast and moulds according to ISO 7954 (1987) and non-lactic-acid bacteria according to a validated in-house colony count technique from Eurofins Food Testing BV, Heerenveen, The Netherlands. Typical sensory aspects such as color, structure, firmness, sweetness, sourness and taste were evaluated by a panel in Uganda in agreement with local practices. Whitening ability and whey separation were also evaluated for the fermented milk products.

### Availability of supporting data

The genome sequence of *S. thermophilus* C106 has been deposited in GenBank with accession number LGRS00000000, BioProject ID PRJNA288538. The genome sequence of *L. rhamnosus* yoba 2012 has been reported previously [8] and deposited at the NCBI BioSample database, Sequence Read Archive, project SRP017797.

## Additional files

10.1186/s12934-015-0370-x Predictive distribution of all functional encoded proteins of *S. thermophilus* C106 into COG Functional Categories: CELLULAR PROCESSES AND SIGNALING: [D] Cell cycle control, cell division, chromosome partitioning; [M] Cell wall/membrane/envelope biogenesis; [N] Cell motility; [O] Post-translational modification, protein turnover, and chaperones; [T] Signal transduction mechanisms; [U] Intracellular trafficking, secretion, and vesicular transport; [V] Defense mechanisms. INFORMATION STORAGE AND PROCESSING (355): [J] Translation, ribosomal structure and biogenesis; [K] Transcription; [L] Replication, recombination and repair. METABOLISM (529): [C] Energy production and conversion; [E] Amino acid transport and metabolism; [F] Nucleotide transport and metabolism; [G] Carbohydrate transport and metabolism; [H] Coenzyme transport and metabolism; [I] Lipid transport and metabolism; [P] Inorganic ion transport and metabolism; [Q] Secondary metabolites biosynthesis, transport, and catabolism and unknown function: [R] Function does not belong to COG categories [S] Function unknown.
10.1186/s12934-015-0370-x Protocol for the production of fermented milk from the seed culture. Front and backside of the sachets designed for African dairy producers. The protocol on the backside indicates two rounds of fermentation: round 1 from the dried bacterial powder (1 g per l) to fermented milk. round 2: from 1 l fermented milk to 50-100 l fermented milk.
10.1186/s12934-015-0370-x Geographical placement of Yoba yoghurt producing groups in Uganda and their weekly volumes as of October 2015. Cities are indicated by blue circles, Yoba producing cooperatives by red circles, and starter culture distribution points by yellow triangles.

## Footnotes

1 The final product of milk fermented by *Lactobacillus rhamnosus* yoba 2012 and *S. thermophilus* C106 as described in this paper is referred to as fermented milk, yoghurt or Yoba.
