# Supplementary material for: A novel consortium of Lactobacillus rhamnosus and Streptococcus thermophilus for increased access to functional fermented foods
Source: Microb Cell Fact. 2015 Dec 8;14:195. doi: 10.1186/s12934-015-0370-x (PMC4672519; doi:10.1186/s12934-015-0370-x)

Additional file 1 Predictive distribution of all functional encoded proteins of *S. thermophilus C106* into COG Functional Categories: CELLULAR PROCESSES AND SIGNALING: [D] Cell cycle control, cell division, chromosome partitioning; [M] Cell wall/membrane/envelope biogenesis; [N] Cell motility; [O] Post-translational modification, protein turnover, and chaperones; [T] Signal transduction mechanisms; [U] Intracellular trafficking, secretion, and vesicular transport; [V] Defense mechanisms. INFORMATION STORAGE AND PROCESSING (355): [J] Translation, ribosomal structure and biogenesis; [K] Transcription; [L] Replication, recombination and repair. METABOLISM (529): [C] Energy production and conversion; [E] Amino acid transport and metabolism; [F] Nucleotide transport and metabolism; [G] Carbohydrate transport and metabolism; [H] Coenzyme transport and metabolism; [I] Lipid transport and metabolism; [P] Inorganic ion transport and metabolism; [Q] Secondary metabolites biosynthesis, transport, and catabolism and unknown function: [R] Function does not belong to COG categories [S] Function unknown.

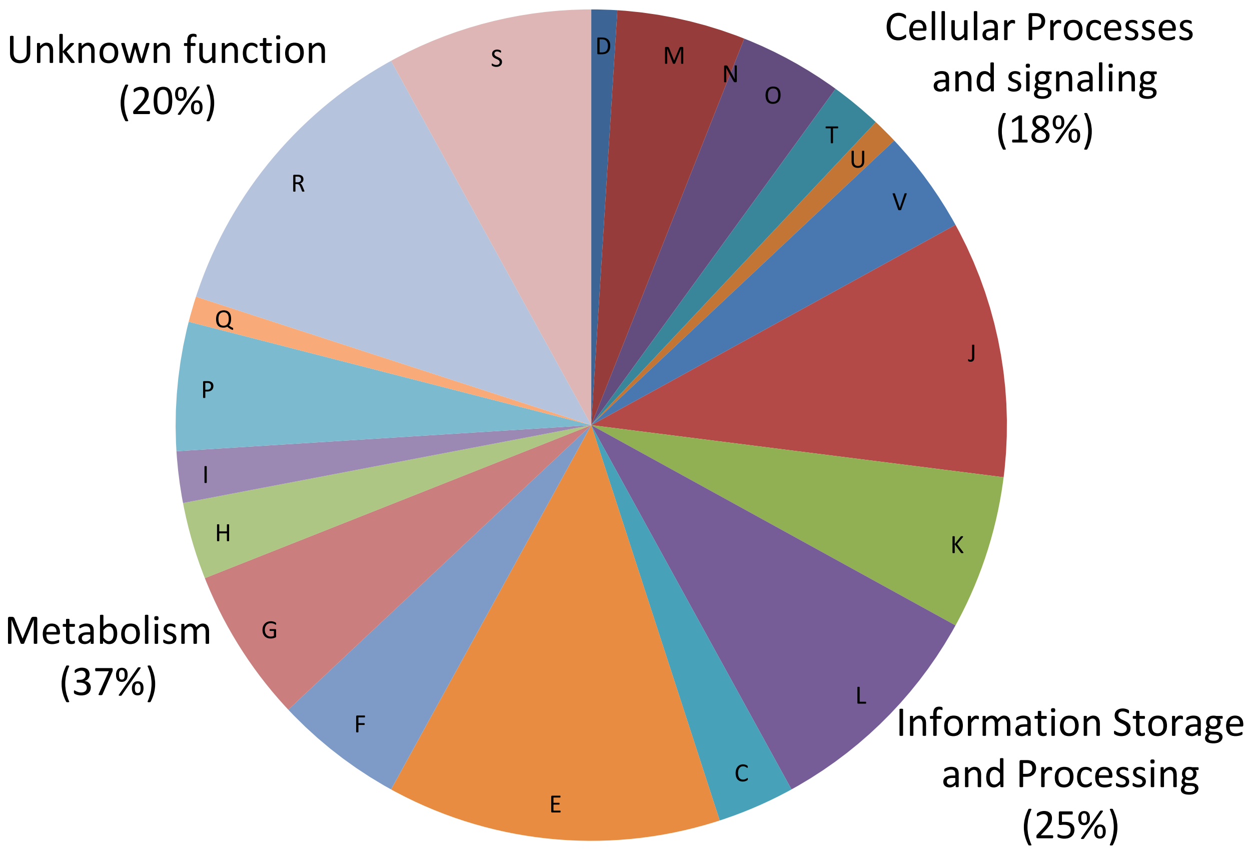

Supplement: Supplementary file 1 — 10.1186/s12934-015-0370-x Predictive distribution of all functional encoded proteins of S. thermophilus C106 into COG Functional Categories: CELLULAR PROCESSES AND SIGNALING: [D] Cell cycle control, cell division, chromosome partitioning; [M] Cell wall/membrane/envelope biogenesis; [N] Cell motility; [O] Post-translational modification, protein turnover, and chaperones; [T] Signal transduction mechanisms; [U] Intracellular trafficking, secretion, and vesicular transport; [V] Defense mechanisms. INFORMATION STORAGE AND PROCESSING (355): [J] Translation, ribosomal structure and biogenesis; [K] Transcription; [L] Replication, recombination and repair. METABOLISM (529): [C] Energy production and conversion; [E] Amino acid transport and metabolism; [F] Nucleotide transport and metabolism; [G] Carbohydrate transport and metabolism; [H] Coenzyme transport and metabolism; [I] Lipid transport and metabolism; [P] Inorganic ion transport and metabolism; [Q] Secondary metabolites biosynthesis, transport, and catabolism and unknown function: [R] Function does not belong to COG categories [S] Function unknown. [file 12934_2015_370_MOESM1_ESM.pdf]
