# Supplementary material for: A novel consortium of Lactobacillus rhamnosus and Streptococcus thermophilus for increased access to functional fermented foods
Source: Microb Cell Fact. 2015 Dec 8;14:195. doi: 10.1186/s12934-015-0370-x (PMC4672519; doi:10.1186/s12934-015-0370-x)

Additional file 2 Protocol for the production of fermented milk from the seed culture.
Front and backside of the sachets designed for African dairy producers. The protocol on the backside indicates two rounds of fermentation: round 1 from the dried bacterial powder (1gram per liter) to fermented milk; round 2 from 1 liter fermented milk to 50-100 liter fermented milk.

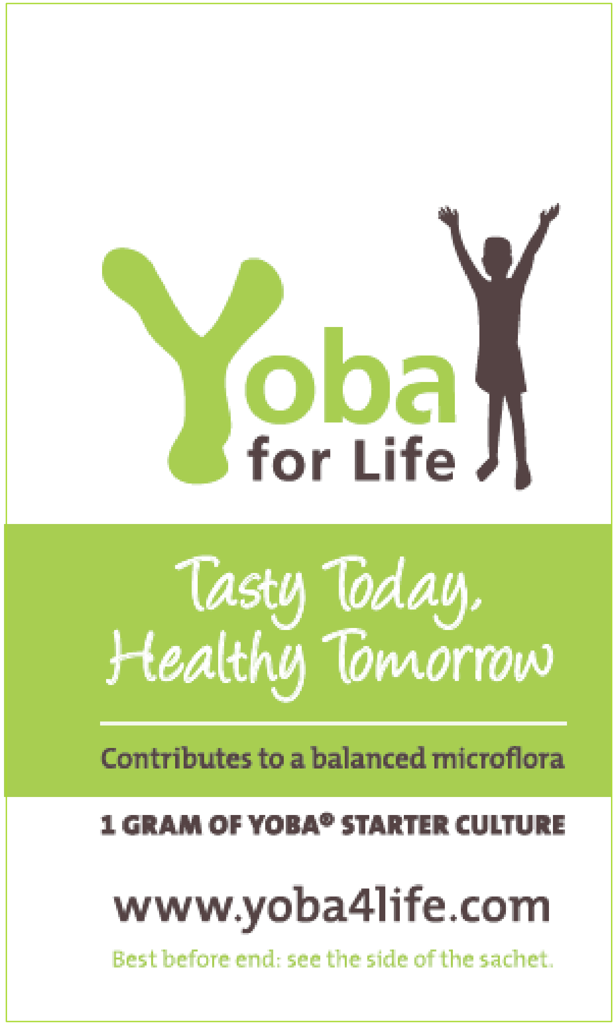

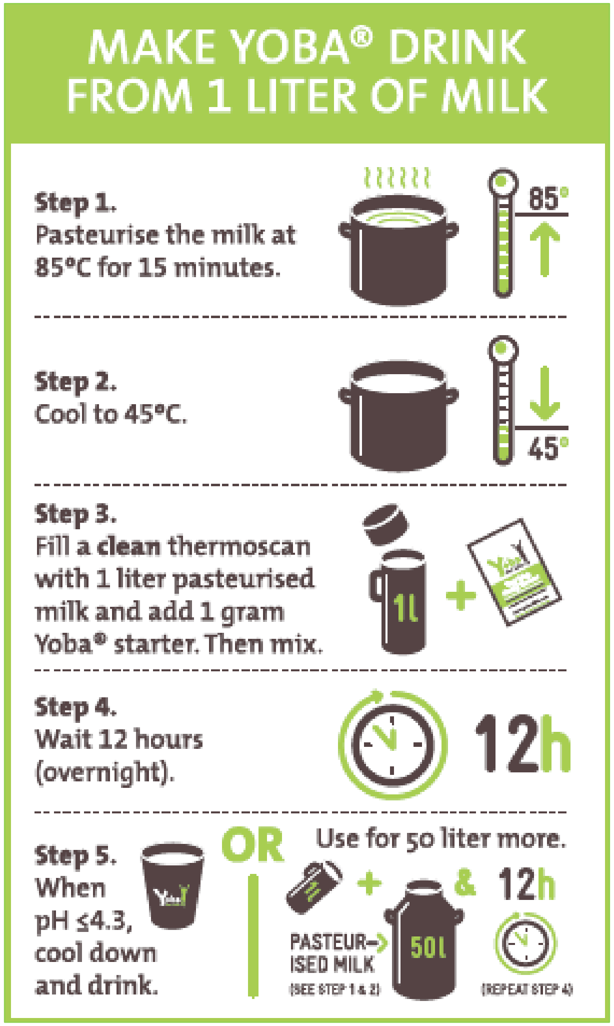

Supplement: Supplementary file 2 — 10.1186/s12934-015-0370-x Protocol for the production of fermented milk from the seed culture. Front and backside of the sachets designed for African dairy producers. The protocol on the backside indicates two rounds of fermentation: round 1 from the dried bacterial powder (1 g per l) to fermented milk. round 2: from 1 l fermented milk to 50-100 l fermented milk. [file 12934_2015_370_MOESM2_ESM.pdf]
