# Supplementary figures and images for: A novel consortium of Lactobacillus rhamnosus and Streptococcus thermophilus for increased access to functional fermented foods
Source: Microb Cell Fact. 2015 Dec 8;14:195. doi: 10.1186/s12934-015-0370-x (PMC4672519; doi:10.1186/s12934-015-0370-x)

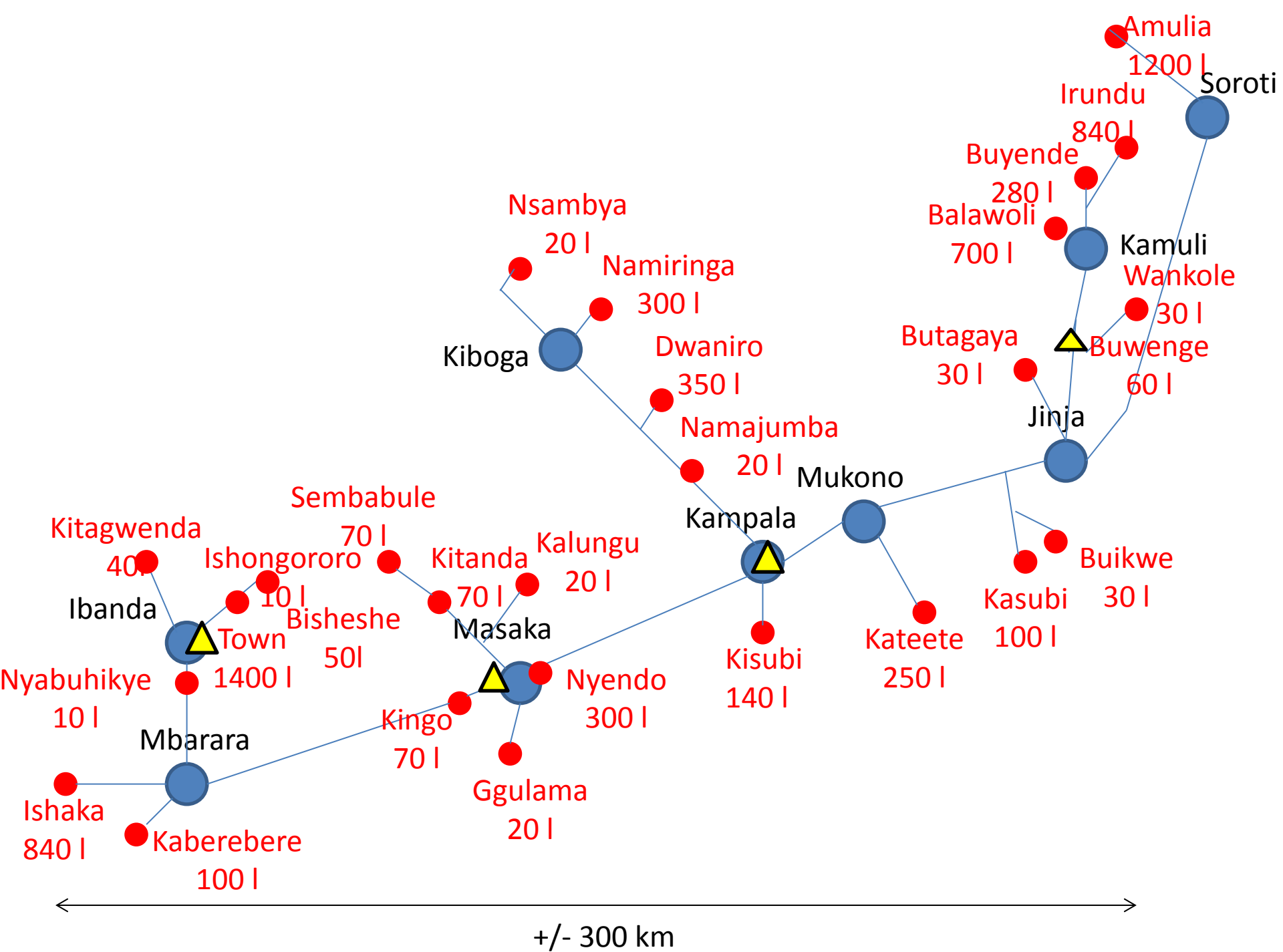

Supplement: Supplementary file 3 — 10.1186/s12934-015-0370-x Geographical placement of Yoba yoghurt producing groups in Uganda and their weekly volumes as of October 2015. Cities are indicated by blue circles, Yoba producing cooperatives by red circles, and starter culture distribution points by yellow triangles. [file 12934_2015_370_MOESM3_ESM.pdf]
